# Characterization of the Fiber Connectivity Profile of the Cerebral Cortex in Schizotypal Personality Disorder: A Pilot Study

**DOI:** 10.3389/fpsyg.2016.00809

**Published:** 2016-05-30

**Authors:** Kai Liu, Teng Zhang, Qing Zhang, Yueji Sun, Jianlin Wu, Yi Lei, Winnie C. W. Chu, Vincent C. T. Mok, Defeng Wang, Lin Shi

**Affiliations:** ^1^Department of Imaging and Interventional Radiology, The Chinese University of Hong KongHong Kong, China; ^2^Department of Radiology, Affiliated Zhongshan Hospital of Dalian UniversityDalian, China; ^3^Department of Psychiatry and Behavioral Sciences, Dalian Medical UniversityDalian, China; ^4^Department of Radiology, The Second People’s Hospital of ShenzhenShenzhen, China; ^5^Shenzhen Research Institute, The Chinese University of Hong KongShenzhen, China; ^6^Department of Medicine and Therapeutics, The Chinese University of Hong KongHong Kong, China; ^7^Research Center for Medical Image Computing, The Chinese University of Hong KongHong Kong, China; ^8^Chow Yuk Ho Technology Centre for Innovative Medicine, The Chinese University of Hong KongHong Kong, China

**Keywords:** schizotypal personality disorder, schizophrenia, diffusion tensor imaging, tractography, brain cortex

## Abstract

Schizotypal personality disorder (SPD) is considered one of the classic disconnection syndromes. However, the specific cortical disconnectivity pattern has not been fully investigated. In this study, we aimed to explore significant alterations in whole-cortex structural connectivity in SPD individuals (SPDs) by combining the techniques of brain surface morphometry and white matter tractography. Diffusion and structural MR data were collected from 20 subjects with SPD (all males; age, 19.7 ± 0.9 years) and 18 healthy controls (all males; age, 20.3 ± 1.0 years). To measure the structural connectivity for a given unit area of the cortex, the fiber connectivity density (FiCD) value was proposed and calculated as the sum of the fractional anisotropy of all the fibers connecting to that unit area in tractography. Then, the resultant whole-cortex FiCD maps were compared in a vertex-wise manner between SPDs and controls. Compared with normal controls, SPDs showed significantly decreased FiCD in the rostral middle frontal gyrus (crossing BA 9 and BA 10) and significantly increased FiCD in the anterior part of the fusiform/inferior temporal cortex (*P* < 0.05, Monte Carlo simulation corrected). Moreover, the gray matter volume extracted from the left rostral middle frontal cluster was observed to be significantly greater in the SPD group (*P* = 0.02). Overall, this study identifies a decrease in connectivity in the left middle frontal cortex as a key neural deficit at the whole-cortex level in SPD, thus providing insight into its neuropathological basis.

## Introduction

Schizotypal personality disorder (SPD) is characterized as a pervasive pattern of social and interpersonal deficits and is diagnosed based on a series of psychotic-like symptoms, including ideas of reference, odd beliefs, and unusual perceptual experiences (American-Psychiatric-Association, 1994; Millon and Davis, 1996; Hazlett et al., 2012b; Rosell et al., 2014). Generally, SPD and schizophrenia share a broad range of similarities in terms of genetics (Kety, 1983; Torgersen, 1985), phenomenology (Raine et al., 1994), neurophysiology (Oestreich et al., 2015b, 2016), and neuroimaging findings (Takahashi et al., 2006; Hazlett et al., 2008a). Therefore, the well-known schizophrenia spectrum theory considers SPD as a milder presentation of pathology along the schizophrenia continuum (Siever and Davis, 2004).

Despite the commonalities, a series of traits were discovered to distinguish SPD from more severe schizophrenia spectrum diseases, and the pattern of neuroanatomical alterations in SPD revealed by magnetic resonance imaging (MRI) was recognized as one of the most significant of those traits (Nakamura et al., 2005). Specifically, the decrease in brain gray matter (GM) in the frontal lobe that is considered a key symptom in schizophrenia is variable in SPD (Rosell et al., 2014), i.e., previous studies of SPD have reported increases (Suzuki et al., 2005; Hazlett et al., 2008a; Kuhn et al., 2012), decreases (Asami et al., 2013), or no difference (Yoneyama et al., 2003) in frontal lobe GM volume. Moreover, compared with schizophrenia, even the common temporal abnormalities were observed to show a more restricted pattern in SPD (Takahashi et al., 2006, 2010, 2011; Hazlett et al., 2008a). Therefore, more evidence is still needed to characterize the pattern of neuroanatomical alteration in SPD, as well as to clarify the underlying basis for the lack of consistency across previous findings.

Compared with the extensive investigation of GM abnormalities, changes in brain white matter (WM) in SPD have rarely been studied. Based on Hazlett’s review (Hazlett et al., 2012b) and our review of the literature, only five studies utilizing MR diffusion tensor imaging (DTI) have described WM abnormalities in SPD (Nakamura et al., 2005; Gurrera et al., 2007; Hazlett et al., 2011, 2012a; Lener et al., 2015). Nevertheless, the findings of these studies and our previous investigation of functional connectivity (Zhang et al., 2014) have consistently suggested that SPD may be one of the classic cortical disconnection syndromes. Therefore, it is of great interest to identify the “disconnection” that occurs in the SPD brain in a comprehensive and exploratory manner. However, most of the currently available WM analysis techniques as used in previous SPD studies cannot provide a direct and comprehensive analysis of whole-cortex connectivity. For example, in region of interest (ROI)-based studies, analyses have been largely dependent on prior hypotheses and confined within several selected WM regions or tracts. Another technique is the tract-based spatial statistics (TBSS) method. Although, this technique is considered a comprehensive whole-brain analysis tool, it may be difficult to use TBSS to directly determine the cortical connectivity because its results are shown in terms of the WM region, which does not directly correspond to the cortical surface.

Therefore, to perform a comprehensive and direct evaluation of cortical connectivity in SPD, a framework for exploratory analysis was designed by combining modern cortical surface reconstruction and diffusion tractography techniques. Briefly, by measuring the number and fractional anisotropy (FA) of the fibers associated with a given unit area of the cortical surface in DTI tractography [i.e., fiber connectivity density (FiCD)], the whole-cortex connectivity can be measured and compared among groups or individuals in a vertex-wise manner (i.e., a data-driven method). In contrast to ROI-based analyses, data-driven approaches analyze the neuroimaging measurement across whole brain on a spatial basis of a vertex (i.e., the smallest unit) and are not dependent on any previously drawn ROI. A data-driven approach can automatically outline the brain regions with statistical significance at a high spatial resolution. Herein, we incorporated this approach into our method to produce a highly automatic, objective, and spatially detailed analytical framework.

In this study, we aimed to use the proposed method to locate the regions with decreases in convergent fiber connectivity in the cortex of SPDs. Moreover, this FiCD mapping framework may provide a unique and appropriate basis for integrating GM and WM findings because the two metrics can be precisely spatially matched in a unified space (i.e., cortical surface). Emerging studies would benefit from a combination of GM and WM metrics, as this may provide novel findings in terms of disease-specific correlative changes in schizophrenia disorders (Koch et al., 2013; Ehrlich et al., 2014). Therefore, further investigation of the relationship between GM and WM abnormalities in the cortex in SPD using this framework would shed new light on the underlying neurobiology.

## Materials and Methods

### Participants

All procedures involving human participants were performed in accordance with the ethical standards of the institutional research committee and the 1964 Helsinki declaration and its later amendments or comparable ethical standards. Informed consent was obtained from all individual participants included in the study. The subjects recruited in this study were screened from a pool of 3000 first-year university undergraduates by using the Minnesota Multiphasic Personality Inventory (MMPI)-566. The criteria for SPD are SC > 70 and 65 < F < 79. Then, 20 SPD individuals (all males; age, 19.65 ± 0.93 years) were selected based on a full diagnostic structured interview for DSM-IV Personality Disorders (Millon and Davis, 1996) by two experienced clinical psychologists. Meanwhile, 18 age- and gender-matched healthy controls (all males; age, 20.33 ± 0.97 years) were included. Moreover, each subject was assessed using the Schizotypal Personality Questionnaire (SPQ; Raine, 1991). None of the SPD patients had previously been hospitalized or used antipsychotic medications. Other exclusion criteria for both groups included any history of other central nervous system disorders or diseases and any contraindication to MR examination. These criteria are also described in our previous report (Zhang et al., 2014).

### MRI Acquisition

Magnetic resonance data were acquired for all subjects using a 3T MR scanner (Siemens, Verio, Germany) equipped with an 8-channel head coil. Three-dimensional T1-weighted images (3D-T1WI) were obtained using a 3D magnetization-prepared rapid acquisition with gradient echo (MPRAGE) sequence with the following parameters: TR/TE/TI = 1900/2.46/900 ms, flip angle = 9°, field of view (FOV) = 250 mm × 250 mm, voxel size = 0.65 mm × 0.65 mm × 1.0 mm. Two diffusion-weighted runs were acquired using echo-planar imaging (EPI) sequences with the following parameters: TR/TE = 8000/93.0 ms, flip angle = 90°, number of excitations (NEX) = 2, FOV = 256 mm × 256 mm, voxel size = 2.0 mm × 2.0 mm × 2.75 mm. Sixty-four diffusion weighting directions with *b* = 1000 s/mm^2^ and two *b*_0_ volumes were obtained for each run.

### Preprocessing

Magnetic resonance data preprocessing was performed in two parts: construction of the cortical GM–WM interface based on 3D-T1WI and preprocessing of diffusion-weighted images (DWIs). Freesurfer (Fischl, 2012)^1^ was used to preprocess 3D-T1WI. The preprocessing steps included intensity normalization, removal of non-brain tissue, automated Talairach transformation, GM–WM segmentation (Fischl et al., 2002), tessellation of the GM–WM boundary, automated topology correction (Segonne et al., 2007), and surface deformation following intensity gradients to optimally place the GM–WM and GM–cerebrospinal fluid border at the location where the greatest shift in intensity defined the transition to the other tissue class (Dale et al., 1999; Fischl and Dale, 2000). Preprocessing of the diffusion data was performed using the FSL toolbox (Jenkinson et al., 2012)^2^. First, head motion and eddy currents in DWI were corrected via affine registration to the first *b*_0_ volume. Then, skull and other non-brain tissues were removed using the Brain Extraction Tool (BET; Smith, 2002) in FSL. Finally, the resultant diffusion images and 3D-T1WI, along with the constructed GM–WM interface of the same subject, were co-registered using the ANTs toolbox^3^.

### Cortical FiCD Mapping

**FIGURE 1:**
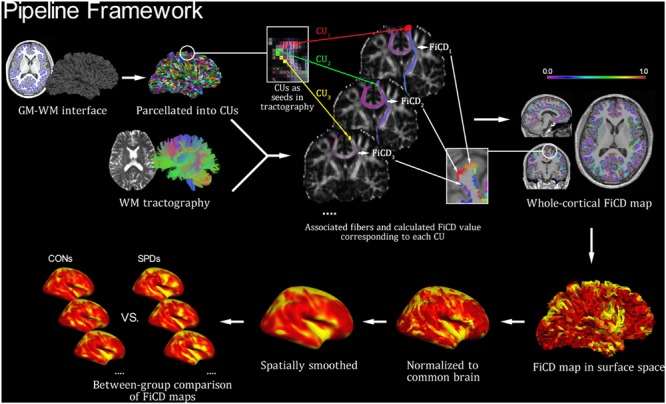
**Pipeline framework of FiCD mapping.** Firstly, both the cortical gray matter–white matter (GM–WM) interface and the WM tractography are constructed, and the GM–WM interface is parcellated to generate 2,000 homogenous cortical units (CUs). Secondly, the CUs are transformed into tractography space and then used as volume seeds to generate their respective fiber sets. The FiCD value for a single CU is calculated based on the properties of its associated fibers, and it is then assigned back to this CU. By repeating this procedure for each CU, a whole-cortex FiCD map is generated. Thirdly, the FiCD map is projected onto surface from volume space, and it is then normalized to the common brain surface and spatially smoothed. Finally, a group-level statistical comparison on the smoothed FiCD maps between schizotypal personality disorder (SPD) and control (CON) groups is performed on the common brain surface.

The FiCD mapping process was performed for each subject according to the following three steps (**Figure 1**). First, the constructed GM–WM interface of the whole cortex was parcellated into 2000 small surface patches [termed cortical units (CUs)], and then the subcortical voxel layer beneath each CU was extracted and used as a segmentation mask (i.e., CU in volume space). Second, each CU in volume space was used as a seed to generate its associated fiber tracks using deterministic fiber tracking (streamline algorithm) in DSI Studio (Yeh et al., 2010, 2013; Yeh and Tseng, 2011) with the following parameters: FA threshold = 0.14, turning angle threshold = 45°, step size = 0.5, smoothing = 0.5, seed number = 1/voxel. In addition, a fiber length constraint of 30–300 mm was used to exclude misidentified fibers. Next, the FA values of all fibers associated with a CU were summed and then divided by the volume of the CU (to correct for the homogeneity of CU size and to highlight the density type). The resulting value was used as the FiCD and assigned back to the corresponding CU. By repeating this step for each CU, a FiCD map of the whole cortex was constructed for an individual brain. Third, the FiCD map of each individual in volume space was projected onto a native brain surface space and then registered to a common surface space in fsaverage via surface registration (Fischl et al., 1999; to ensure an accurate alignment of the gyral structures among individuals). Finally, after spatial smoothing with a 10 mm full-width at half-maximum (FWHM) Gaussian kernel, a vertex-wise inter-group statistical analysis was performed in the common surface space. Briefly, after the surface registration, each vertex on the cortical surface was spatially coregistered across all the subjects in the common surface space. Thus, the statistical comparisons of the FiCD values could be performed between the two groups vertex-by-vertex across the whole cortex.

### Group-Level Statistical Analysis

Vertex-wise comparison of the FiCD maps between the SPD and control (CON) groups was tested using a general linear model (GLM) with a significance threshold of *P* = 0.05 (Monte Carlo Null-Z simulation was used to correct for multiple comparisons, with 10,000 iterations and an inclusion threshold of *P* = 0.05). To increase the normality before the inter-group statistical analysis, the FiCD of each vertex was transformed by subtracting the mean value and then dividing by the standard deviation of the whole-hemisphere FiCD (i.e., a *Z*-transform). After the group comparison, the mean FiCD values and GM volume values of clusters with significant between-group differences were extracted for each subject and used for subsequent ROI-based analysis. Partial correlation to control for total brain volume was used to evaluate the relationships between SPQ score and FiCD, between SPQ score and GM volume, and between FiCD and GM volume in each significant cluster, with a significance threshold of *P* = 0.05. Moreover, the GM volume values of the significant clusters were also compared between groups using two-sample *t*-tests with a significance threshold of *P* = 0.05. The between-group differences in age and total brain volume were analyzed using two-sample *t*-tests (or Mann–Whitney *U*-test if the data were not normally distributed) with a significance threshold of *P* = 0.05.

### Fiber Tracking Analysis based on Cortices with Differences in FiCD

To further investigate whether the observed between-group differences in FiCD in the previous step were driven by changed fiber number (FN) or WM integrity, we also performed the following fiber tracking analyses. First, the cluster(s) identified with significant between-group differences in FiCD on the common cortical surface space were inversely transformed to the individual cortical surface of each subject. Second, the subcortical voxel layer beneath the area of the cluster(s) was extracted and used as a segmentation mask (i.e., seed) to perform fiber tracking in the tractography of each subject. Third, the total number and mean FA value of the fibers generated from the cluster seed were calculated and then compared between the SPD and CON groups with a significance threshold of *P* = 0.05.

Moreover, fibers connecting to clusters with connectivity decreases were visualized using common brain WM tractography to show the WM pathways potentially affected by the disease. The normal brain DTI template of the Illinois Institute of Technology (IIT; Zhang et al., 2011) in Montreal Neurological Institute (MNI)-152 space was used as a common brain WM reference. Briefly, the cortical clusters with significant FiCD decreases identified in previous between-group comparisons were transformed to MNI-152 volume space and then used as seeds to generate the associated fiber tracts based on the IIT tractography. Thus, the generated fiber tracks represented the normal WM fibers connecting to the cortical area of interest and corresponded anatomically to the fiber pathways linking to the cortex showing disconnection in the SPD group.

## Results

### Sample Characteristics

There was no significant difference in total brain volume (*P* = 0.13) or total WM volume (*P* = 0.40) between the SPD and control groups. The SPD group showed significantly (*P* < 0.001) higher SPQ scores (32.0 ± 15.0) compared with the controls (13.3 ± 7.7).

### Vertex-Wise Differences in the FiCD Maps between SPD and Controls

**FIGURE 2:**
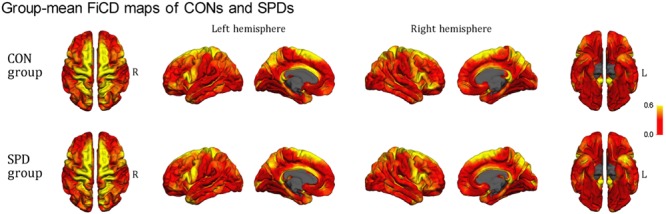
**Mean FiCD maps of the SPD and CON groups.** A symmetrical distribution of FiCD on cortex between the left and right hemispheres is shown. In both groups, cortical regions with high FiCD value are found in regions including the cingulate gyrus, precentral gyrus, superior frontal gyrus, pre- and post-central gyri, pars opercularis, superior and inferior parietal gyri, precuneus, the anterior part of the temporal lobe, and the parahippocampal gyrus.

**FIGURE 3:**
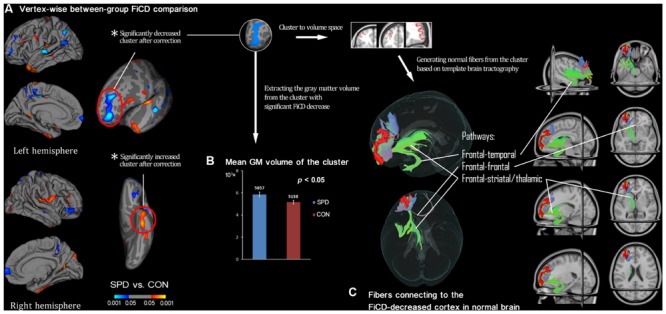
**Between-group comparison of SPDs vs. CONs and subsequent ROI-analysis on cortex with decreased connectivity. (A)** Vertex-wise between-group comparison shows decreased/increased FiCD value of SPD compared with control (CON) group in blue/red (*P* < 0.05, uncorrected). After Monte Carlo simulation correction, only two clusters survived, and they are marked with red circles. **(B)** The extracted GM volume from the cluster with FiCD decrease is significantly larger in the SPD group. **(C)** The significant cluster is transformed into the volume space of the common brain (MNI-152), and it is then used as a seed to perform fiber tracking. Thus, the fiber pathways in normal brain connected to the cortical region corresponding to the cluster with significantly decreased connectivity are identified and illustrated.

**Figure 2** illustrates the respective mean FiCD maps for the SPD and CON groups. In both groups, cortical regions with comparatively high FiCD value were found in regions including the cingulate gyrus, precentral gyrus, superior frontal gyrus, pars opercularis, and precuneus. Moreover, the cortical FiCD showed a general symmetrical distribution between the left and right hemispheres. Compared with that of the CON group, the rostral middle frontal gyrus (crossing BA 9 and BA 10) of the SPD group showed significantly decreased FiCD (**Figure 3A**). A significantly increased FiCD was found in the SPD group in the anterior part of the fusiform/inferior temporal cortices (*P* < 0.05, Monte Carlo simulation corrected). No cluster in the right hemisphere was found to be significant after correction.

### ROI-Based Analysis of Significant Cortical Clusters

The mean FiCD values extracted from the above two clusters with significant between-group differences were not significantly correlated with the individual SPQ score in either SPD or CON groups, while an insignificant tendency of positive correlation was observed in the SPD group between the SPQ score and the mean FiCD of the left middle frontal cluster (*R* = -0.44, *P* = 0.06). A significant correlation was found between the SPQ score and the extracted GM volume of the middle frontal cluster in the SPD group (*R* = -0.50, *P* = 0.03), while the correlation between GM volume and SPQ was non-significant in the temporal cluster for both groups. No significant correlation was found between the FiCD values and the GM volumes of the two clusters in either group. All the correlation results are summarized in **Table 1**. The between-group comparison of the GM volume from the two significant clusters revealed a significantly increased GM volume of the middle frontal cluster in SPDs (*T* = 2.531, *P* = 0.02, **Figure 3B**), whereas the increase in the temporal cluster was not significant (*T* = 1.828, *P* = 0.08).

**Table 1:** Correlation results among schizotypal personality questionnaire (SPQ) score, fiber connectivity density (FiCD) value, and gray matter (GM) volume of the two clusters with significant FiCD difference between schizotypal personality disorder (SPD) and control (CON) groups.

	SPD group (*n* = 20)		CON group (*n* = 18)	
	*R*-value	*P*-value	*R*-value	*P*-value
SPQ and FiCD (C1↓)	-0.44	0.06	0.22	0.39
SPQ and FiCD (C2↑)	-0.37	0.12	-0.09	0.73
SPQ and GM volume (C1)	-0.5	0.03^∗^	0.28	0.28
SPQ and GM volume (C2)	0.02	0.94	0.18	0.49
FiCD (C1↓) and GM volume (C1)	0.38	0.11	-0.15	0.58
FiCD (C2↑) and GM volume (C2)	-0.01	0.98	-0.13	0.61

*FiCD (C1↓): the mean FiCD value of the cluster with significantly decreased FiCD, i.e., the middle frontal cluster; FiCD (C2↑): the mean FiCD value of the cluster with significantly increased FiCD, i.e., the temporal cluster; GM volume (C1): the gray matter volume of the middle frontal cluster; GM volume (C2): the gray matter volume of the temporal cluster; All the correlations were controlled for total brain volume; ^∗^ indicates a statistically significant *P*-value.*

### Fiber Tracking Analysis on Cortices with Changed FiCD

By performing fiber tracking using the two significant clusters as seeds, the fibers connecting to each cluster were generated for each subject. Between-group comparison revealed that both FA and FN of SPDs were reduced in the frontal cluster (FA: *T* = -1.327, *P* = 0.19; FN: *T* = -2.099, *P* = 0.04) and elevated in the temporal cluster (FA: *T* = 0.725, *P* = 0.47; FN: *T* = 4.385, *P* < 0.001). However, only the differences in FN showed statistical significance.

By using the cluster with significant FiCD decrease as the seed (i.e., the middle frontal cluster, **Figure 3C**), fiber tracking in common brain space revealed the following four major pathways: (1) the fronto-temporal pathway, connecting to the ipsilateral temporal lobe; (2) the fronto-striatal pathway, connecting to the striatum (including the ipsilateral caudate nucleus, putamen, and globus pallidus); (3) the fronto-thalamic pathway, connecting to the ipsilateral thalamus; and (4) the fronto-frontal pathway, connecting to the contralateral frontal cortex via the genu of the corpus callosum.

## Discussion

In this study, we used a new analytical framework to comprehensively explore the abnormalities of cortical connectivity density in individuals with SPD. There were three major findings. First, group-level and whole-cortex-level alterations in convergent fiber connectivity were observed in the left middle frontal gyrus and the left inferior temporal lobe in the SPD group. Second, the change in GM volume of the cortex observed along with the connectivity decrease was correlated with the severity of disease (i.e., SPQ score). Third, the cortical region that demonstrated a connectivity decrease also demonstrated a GM morphometric abnormality.

### Decreased Fiber Connectivity Density of the Middle Frontal Gyrus in SPD

One of the most significant findings of the current study was the decrease in FiCD observed in the rostral middle frontal gyrus (crossing BA 9 and BA 10) in SPD. Although previous DTI studies on SPD are limited, there is evidence that directly supports this finding. Among the five previous DTI studies, four identified significant changes in diffusion indices in the WM regions or WM tracts connecting with the prefrontal cortex (Nakamura et al., 2005; Gurrera et al., 2007; Hazlett et al., 2012a; Lener et al., 2015). According to Hazlett et al. (2012a), individuals with SPD demonstrated fewer fiber tracts projecting to BA 10 (rather than BA 45) from the anterior limb of the internal capsule. Moreover, in Lener’s investigation (Lener et al., 2015), a whole-brain exploratory analysis (i.e., TBSS) revealed an SPD-related abnormality that was found only in the genu of the corpus callosum (fibers connecting the left and right prefrontal cortices). Therefore, when considered with our current findings, this evidence highlights the role of abnormal prefrontal connectivity (especially in BA 10) in SPD. In addition, regarding the restriction of the connectivity change to the left hemisphere, our result may be in line with previous meta-analyses findings in schizophrenia (Wright et al., 2000; Buchsbaum et al., 2006; Ellison-Wright and Bullmore, 2009). These findings have shown that the left frontal and temporal WM fibers appear to be affected more frequently, perhaps suggesting a disease-specific alteration pattern of brain connectivity in schizophrenia. However, additional evidence is still necessary to further confirm the laterality issue in SPD because the currently available studies are quite limited. Furthermore, when investigating the factors that lead to the observed differences in cortical connectivity, the role of FN (instead of FA) is highlighted. On the one hand, it may indicate that the altered cortical connectivity in SPD is primarily driven by the number of fibers, while the integrity of fibers is comparatively preserved. On the other hand, this observation may also be influenced by the age range of our cohort in that the subjects in both groups were approximately 20 years-old and basically free from WM degeneration or injuries.

### Lower Fiber Connectivity but Greater GM Volume in the Middle Frontal Gyrus in SPD

Compared with the convergent previous evidence of prefrontal impairment in schizophrenia, neuroanatomical findings regarding the prefrontal lobe in SPD are relatively inconsistent, leading to the proposal of the frontal sparing hypothesis (Buchsbaum et al., 2002; Hazlett et al., 2008a, 2012b; Rosell et al., 2014). This hypothesis is based on a large number of GM morphometry findings and states that a general preservation of the frontal lobe (especially BA 10) in SPD compared with schizophrenia can be attributed to a neural ‘protective factor’ against the psychosis phenotype. Interestingly, in this study, we found that in SPDs, the middle frontal gyrus simultaneously showed decreased fiber connectivity and increased GM volume. On the one hand, our results support previous findings of an increase in GM volume (Suzuki et al., 2005; Hazlett et al., 2008a; Kuhn et al., 2012) in the prefrontal cortex of SPD and are also consistent with increased metrics of the prefrontal regions of SPDs in a variety of other modalities, e.g., higher metabolic rates related to verbal learning in BA 10 shown using fluorodeoxyglucose-Positron emission tomography (FDG-PET; Buchsbaum et al., 2002) and higher activation in BA 9 related to prepulse inhibition using functional MRI (fMRI; Hazlett et al., 2008b). On the other hand, we observed that the GM volume of the prefrontal cluster in SPD, though increased compared with healthy controls, decreased as the disease became more severe. This finding may lead us to the hypothesis that the increased prefrontal GM may be a disease-stage-dependent process that may become exhausted as the disease severity increases. Although this speculation is highly hypothetical, it may be further supported by our results showing opposite directions of correlations between GM volume and SPQ, respectively, observed in the CON group (with a tendency toward positive correlation) and in the SPD group (showing a negative correlation). Moreover, this correlation pattern is also consistent with previous studies (Hazlett et al., 2008a; Asami et al., 2013) and may help explain the lack of consistency across studies regarding the direction of altered GM volume in SPD.

When considering the non-significant correlation between GM volume and WM connectivity in the middle frontal cortex, we may conclude that the cortical GM increase is probably not induced by (and probably does not compensate for) the corresponding subcortical WM disconnection in the same region, as hypothesized for SPD by a previous study (Hazlett et al., 2012a). Nevertheless, it may be interesting to note that the tendency of GM–WM correlation in the frontal cluster was shifted from a negative correlation in control group to a positive correlation in SPD group. From the developmental perspective, in healthy individuals, a cortical GM reduction could be closely accompanied by increasing myelination around the GM–WM interface during brain maturation and the development of cognitive abilities (Giorgio et al., 2008; Tamnes et al., 2010), possibly reflecting an adaptive neural-shaping process of synaptic pruning and age-related reduction in neuropil (Huttenlocher and Dabholkar, 1997). In contrast, due to deterioration of both GM and WM, this development-related negative association between GM and WM may be different in schizophrenia (Ehrlich et al., 2014). Thus, the current tendency of a reversed GM–WM association pattern in SPD may be consistent with that in schizophrenia and may be interpretable as a developmental aberration (Siever and Davis, 2004). However, it is important to note that this reversed association was not statistically significant but was only a tendency based on our small cohort. Therefore, future investigations are still encouraged to further reveal the disease-specific correlative changes between GM and WM in SPD.

### Increased Fiber Connectivity Density of the Inferior Temporal Region in SPD

The other significant finding of this study was an increased FiCD in the fusiform and inferior temporal region (anterior part). Although, most studies have recognized decreased measurements in the temporal lobe (especially the superior temporal gyrus) as a morphological substrate of SPD, some previous evidence may help to explain and support our present results. According to Takahashi’s study (Takahashi et al., 2006), which focused on a morphometric analysis of the temporal lobe, a decrease in volume of the fusiform gyrus in SPD was localized only in the posterior part, whereas the left anterior fusiform actually showed a tendency toward a GM increase (5563 vs. 5287 mm^3^, not statistically significant) in their male subjects. Moreover, the increased WM volume in the inferior temporal gyrus identified by Hazlett et al. (2008a) may support our finding more directly.

### Neural Pathways Potentially Involved in SPD

The fiber-tracking results of this study are highly consistent and correspond with previous findings in that each of the identified pathways connecting to the cortex with fiber connectivity decreases was described by most of the previous DTI studies on SPD (Nakamura et al., 2005; Gurrera et al., 2007; Hazlett et al., 2011, 2012a; Lener et al., 2015) and was considered to be significant. First, the fronto-temporal disconnectivity in the uncinate fasciculus (UF) is among the earliest and best-known WM abnormalities in SPD (Nakamura et al., 2005; Gurrera et al., 2007). Meanwhile, it is worth mentioning that healthy individuals with schizotypal features have been reported to show increased FA values in the UF (Smallman et al., 2014). Thus, it may be highly interesting in the future to investigate how the WM status of the UF changes along the psychosis/schizophrenia continuum and how it may be related to different disease phenotypes. Second, decreases in both frontal-striatal (Suzuki et al., 2004; Hazlett et al., 2012a) and frontal-thalamic (Oh et al., 2009; Hazlett et al., 2012a) connectivities are well-documented. It should be noted that an aberrant frontal-striatal pathway might be consistent with changes in dopaminergic activity in individuals with SPD (Shihabuddin et al., 2001; Levitt et al., 2002; Siever and Davis, 2004) and thus was considered to be associated with psychotic and deficit-like symptoms (Siever and Davis, 2004). Third, the genu of the corpus callosum, which is responsible for connecting the left and right frontal cortices, showed a significant decrease in integrity at the whole-brain level (Lener et al., 2015). Therefore, these fiber-tracking findings further explain and validate the significance of our results of a FiCD decrease in the prefrontal cortex of individuals with SPD.

### Limitations

Several limitations of this study should be noted. First, similar to many previous studies on SPD, the sample size was limited. This might influence the power of the statistical analyses and may have explain the insignificance of some correlations of interest (e.g., the correlation between SPQ and FiCD in the middle frontal cluster, which had a *P*-value of 0.06). For the same reason, a significance threshold of a *P*-value of 0.05 was adopted for both vertex-wise and ROI-based analyses. Although, this may be an acceptable threshold considering the preliminary nature of the current study, it might be less stringent than would provide highly reliable conclusions. In this context, the results should be interpreted with caution. Second, similar to traditional DTI analysis methods, the FiCD framework includes a series of adjustable parameters (e.g., fiber tracking algorithms, anisotropy threshold, and ROI size). Thus, it should be noted that these parameters may influence the results. Third, this study only included male Chinese SPD subjects because of the between-gender distinction in SPD pathophysiology (Siever and Davis, 2004) and differences in language-related WM properties previously noted in schizophrenia (Oestreich et al., 2015a). Thus, future FiCD investigations concerning female and Caucasian individuals with SPD, as well as schizophrenia, are encouraged because they would provide more comprehensive findings and would better address the topic of the schizophrenia spectrum.

In summary, this study explored changes in whole-cortex fiber connectivity in SPD. The results revealed significantly decreased FiCD connecting to the rostral middle frontal cortex (crossing BA 9 and BA 10) in SPD, which might reflect a reduced structural connectivity in this region. Moreover, an accompanying GM volume increase in the same area is also observed in SPD. The present findings may provide new insight into the neurobiological basis of SPD, particularly from the perspective of cortical connectivity, and enrich our understanding of the schizophrenia spectrum disorders.

## Author Contributions

KL and TZ: analyzed the data and wrote the manuscript; QZ, YS, and JW: recruited the subjects and collected MRI data; YL, WC, VM, DW and LS: assisted in the study design and manuscript preparation and supervised the study; LS: performed final approval of the version to be published.

## Conflict of Interest Statement

The authors declare that the research was conducted in the absence of any commercial or financial relationships that could be construed as a potential conflict of interest.

## Footnotes

1 http://surfer.nmr.mgh.harvard.edu

2 http://www.fmrib.ox.ac.uk/fsl/

3 http://www.picsl.upenn.edu/ANTS/
